# Integrated GC–MS- and LC–MS-Based Untargeted Metabolomics Studies of the Effect of Vitamin D3 on Pearl Production Traits in Pearl Oyster *Pinctada fucata martensii*

**DOI:** 10.3389/fmolb.2021.614404

**Published:** 2021-03-05

**Authors:** Chuangye Yang, Yetao Zeng, Yongshan Liao, Yuewen Deng, Xiaodong Du, Qingheng Wang

**Affiliations:** ^1^ Fisheries College, Guangdong Ocean University, Zhanjiang, China; ^2^ Guangdong Science and Innovation Center for Pearl Culture, Zhanjiang, China; ^3^ Guangdong Provincial Engineering Laboratory for Mariculture Organism Breeding, Zhanjiang, China; ^4^ Pearl Breeding and Processing Engineering Technology Research Centre of Guangdong Province, Zhanjiang, China

**Keywords:** metabolomics, vitamin D3, *Pinctada fucata martensii*, pearl production traits, GC–MS, LC–MS

## Abstract

Pearl oyster *Pinctada fucata martensii* is widely recognized for biomineralization and has been cultured for high-quality marine pearl production. To ascertain how dietary vitamin D3 (VD3) levels affect the features of pearl production by *P. f. martensii* and discover the mechanisms regulating this occurrence, five experimental diets with variable levels of VD3 were used with inclusion levels of 0, 500, 1,000, 3,000, and 10,000 IU/kg. The distinct inclusion levels were distributed into five experimental groups (EG1, EG2, EG3, EG4, and EG5). All the experimental groups were reared indoors except the control group (CG) reared at the sea. Pearl oysters, one year and a half old, were used in the grafting operation to culture pearls. During the growing period that lasted 137 days, EG3 had the highest survival rate, retention rate, and high-quality pearl rate. A similar trend was found for EG3 and CG with significantly higher pearl thickness and nacre deposition rates than other groups, but no significant differences were observed between them. A metabolomics profiling using GC–MS and LC–MS of pearl oysters fed with low quantities of dietary VD3 and optimal levels of dietary VD3 revealed 135 statistically differential metabolites (SDMs) (VIP > 1 and *p* < 0.05). Pathway analysis indicated that SDMs were involved in 32 pathways, such as phenylalanine metabolism, histidine metabolism, glycerophospholipid metabolism, alanine aspartate and glutamate metabolism, arginine and proline metabolism, glycerolipid metabolism, amino sugar and nucleotide sugar metabolism, and tyrosine metabolism. These results provide a theoretical foundation for understanding the impacts of VD3 on pearl production traits in pearl oyster and reinforce forthcoming prospects and application of VD3 in pearl oyster in aquaculture rearing conditions.

## Introduction

Biomineralization is widely distributed into the marine environment and essential to the welfare of several organisms. The pearl oyster *Pinctada fucata martensii* is widely recognized in biomineralization. It is a filter feeder and a representative species for producing high-quality marine pearl in China and Japan, accounting for more than 90% of seawater pearl produced (Southgate and Lucas, 2008; Z. F. Gu et al., 2009). The traditional method for culturing *P. f. martensii* is at the open sea in a floating raft or a vertical pile exposing them to variable and sometimes extreme environmental conditions (Adzigbli et al., 2020). This susceptibility has caused a decreasing trend in pearl yield since the 1990s due to mass mortality and deteriorating environments (Miyazaki et al., 1999; He et al., 2008; Qiu et al., 2014). This decrease has influenced the pearl industry negatively. The aforementioned disadvantages can be avoided through land-based culturing; however, high dietary demand and the limited information on nutrition requirements for bivalves are some of the big challenges for land-based cultures (Yang et al., 2019a).

Basic knowledge of the essential requirements and correct formulation on artificial diets has big impacts on animal performance. Therefore, an effective diet formulation is essential and demanding in pearl oyster culture. Vitamin D (VD) is a secosteroid involved in physiological functions in several organisms. It is well known for the impact in aquatic organisms’ immunity but also has a key role in regulating calcium and phosphorus metabolism and bone development (Cerezuela et al., 2009). VD3 can stimulate the absorption of calcium by the small intestine and kidneys and promote the synthesis and secretion of osteocalcin by osteoblasts to directly stimulate bone formation, provide the necessary raw materials for normal bone tissue mineralization, and participate in the metabolic control of bone development and resorption (J.H. Gu et al., 2009). Hence, VD3 is an essential nutrient for the standard growth and development of aquatic organisms. An appropriate amount of VD3 in the feed can promote the deposition of minerals in the shell, whereas lack or excessive supplementation of VD3 can inhibit the accumulation of calcium in the shell (Zhou and Mai, 2004). Optimal concentrations of VD3 in feed need to be carefully considered and assessed, particularly for species that are not extensively studied.

Animals will reach different metabolic states with different quality diets. Metabolomics has been proposed to be a model for metabolic studies (Cappello et al., 2017; Cappello et al., 2018; Hao et al., 2018a), to assess metabolic responses to nutritional deficiencies or excesses providing in-depth mechanistic insights to assist the development of optimized feeding regimes (Alfaro and Young, 2018; Hao et al., 2018a). Being an evolving technology and analytical tool, metabolomics is widely used to quantify low-molecular-weight metabolites using distinct tools categorized by the type of instrumental data and the functionality such as nuclear magnetic resonance (NMR), gas chromatography coupled to mass spectrometry (GC–MS), and liquid chromatography coupled with single-stage mass spectrometry (LC–MS) (Alfaro and Young, 2018; Venter et al., 2018; Zhang et al., 2021; Yang et al., 2020; Young and Alfaro, 2018). Exploring this approach in evaluating impacts of limited food, nutrient being supplemented, modifications in nutrient quantities, and replacing protein or lipid diets in aquatic animals has been conducted (Alfaro and Young, 2018; Yang et al., 2018b).

Consequently, the primary aim of this study was to investigate distinct diets varying in the VD3 levels given to pearl oyster cultured in a land-based facility after nucleus operation. This was measured by GC–MS and LC–MS during pearl culture. This study will additionally influence the full comprehension of the principal phenomenon of VD3 in modulating metabolites on pearl oyster and extend the production of *P. f. martensii* in captivity.

## Materials and Methods

### Rearing and Experimental Diets

Organisms were fed one of five experimental treatments, consisting of different dietary levels of VD3 (0, 500, 1,000, 3,000, and 10,000 IU/kg) according to Yang et al. (2019b) and the feeds were kept at −20°C until use.

The research was performed from October 2018 to March 2019. The fifth-generation line, “Haixuan NO 1” with enhanced growth, was utilized for the study. Pearl oysters aged 1.5 years old with shell length of 60.35 ± 6.11 mm in average were selected for the grafting operation. During the operation, a mantle piece (about 4 mm^2^) from a donor oyster and a nucleus (5.5 mm in diameter) were inserted into the host oyster’s gonad according to conventional methods (He et al., 2020). The organisms were randomized to the EG1, EG2, EG3, EG4, and EG5 and a control group (CG). CG was grown in a commercial farm (20°25′N, 109°57′E) at Liusha Bay, Guangdong, China. EGs were grown in tanks with three tanks allocated for individual groups. Every tank was filled with water (1000 L) with seven net cages, and 30 organisms were allocated to individual cages. The amount of diet to every tank was quantified according to previous studies (Wang et al., 2016), and feeding was performed every 4 h. The daily renovation rate of the water was at 300 L. The experiment lasted 137 days under controlled environmental conditions with salinity at 30 ppt, temperature between 18.3° and 30.5°C, and dissolved oxygen at 5.00 mg/L.

### Survival Rate and Pearl Trait Measurement

At the end of the experimental period, the cultured pearls were harvested. A comparison of the survival rate, retention rate, and pearl thickness of the groups was conducted. The survival rate is expressed as the number of oysters that survived in individual replicates to that of individual replicates at the commencement of the study. The retention rate was expressed as the number of harvested pearls in the replicate to that of surviving oysters in the replicates at the end of the study. Measuring the thickness of pearls was accomplished with Optical Coherence Tomography OSG-1000 (Shenzhen MOPTM Imaging Technique Co., LTD., China) according to procedures defined by Li et al. (2018). Nacre deposition rates were evaluated according to procedures defined by Muhammad et al. (2020). High-quality pearl rate is expressed as the number of high-quality pearls in the replicates to that of harvested pearls after the study. High-quality pearls are those showing luster devoid of any circle and calcitic defect on the surface (Le Luyer et al., 2019).

### Metabolomics Sampling

After the 137 days’ culture period, a total of 16 hepatopancreas were collected from each group, instantly preserved in liquid nitrogen, and then stored at −80°C for later analysis.

### GC-TOF-MS Analysis

#### Metabolites Extraction for GC-TOF-MS Analysis

Samples of 50 ± 1 mg were moved into 2 ml vials. Upon the addition of 1 ml of extract solvent (acetonitrile: methanol: water = 2:2:1), including internal standard (2-chloro-L-phenylalanine, 2 μg/ml), samples were homogenized for 30 s using a vortex and with a ball mill for 4 min at 45 Hz. Then samples were ultrasonicated for 5 min in ice and centrifuged at 4°C, 15 min, 10,000 rpm followed by a transfer of 300 μL supernatant to a fresh tube. For the QC (quality control) preparation, 50 μL of each sample was taken and they were combined together. Upon evaporation in a vacuum concentrator, 40 μL of methoxyamination hydrochloride (20 mg/ml in pyridine) was added for incubating at 80°C for 30 min, followed by derivatization with 60 μL of bis-(trimethylsilyl)-trifluoroacetamide regent (1% trimethylchlorosilane, v/v) at 70°C during 90 min. The last step was a progressive cooling at 37°C and addition of 5 μL of saturated fatty acid methyl ester (in chloroform) to the QC sample. Using a gas chromatograph together with a time-of-flight mass spectrometer (GC-TOF-MS), the samples were further analyzed.

#### GC-TOF-MS Analysis

The GC-TOF-MS analysis was conducted using an Agilent 7890 gas chromatograph and a time-of-flight mass spectrometer. The carrier gas was helium and the equipment uses a capillary column (DB-5MS). The settings were in the spitless mode and 1 μL aliquot of the sample was used to run the analysis. The gas flow rate across the column was 1 ml/min and the purge flow of the front inlet was 3 ml min^−1^. At startup, the temperature was kept at 50°C for 60 s, before increasing to 310°C at a rate of 10°C/min, and it was kept again at 8 min for 310°C. The ion source temperatures, transfer line, and injection were established at 250°C, 280°C, and 280°C, respectively. At the electron impact mode, the energy was established at −70 eV. After a solvent delay of 6.33 min, the mass spectrometry was obtained using a full-scan with a range of 50–500 at a speed of 12.5 spectra/second.

#### Data Preprocessing and Annotation for GC-TOF-MS Analysis

The deviation value was filtered to remove noise by interquartile range for single data method. A single peak was then filtered and the peak area data were retained with the null value of a single group, not N50%, or the null value of all groups, not N50%. The missing values of the original data were filled with the minimum 1/2 method and the data was normalized using the internal standard method to obtain the relative quantitative value. The raw data analyses, such as baseline adjustment, peak extraction, alignment, deconvolution, and integration, were performed using Chroma TOF (V 4.3x, LECO) software. The LECO-Fiehn Rtx5 database was used in the identification of metabolites calculating the retention index and the mass spectrum (Kind et al., 2009). Retention index match and mass spectrum match were considered in metabolite identification. The retention time index (RI) method was used for peak identification, and the RI tolerance was 5,000. The peaks detected in <½ of QC samples or Relative Standard Deviation (RSD) > 30% in the QC samples were discarded (Dunn et al., 2011). GC–MS-based metabolomics raw data were in Supplementary Data Sheet S1.

### LC-TOF-MS Analysis

#### Metabolite Extraction for LC-TOF-MS Analysis

50 mg of sample was weighted to an Eppendorf tube, and 1,000 μL extract solution (acetonitrile: methanol: water = 2: 2: 1) containing internal standard (L-2-chlorophenylalanine, 2 μg/ml) was added. After vortexing the samples for 30 s and homogenizing at 35 Hz for 4 min, sonication of samples was performed for 5 min in an ice-water bath. The homogenization and sonication procedure were performed twice. Incubation and centrifugation were then performed at −40°C for 60 min and 10,000 rpm, 15 min at 4°C, respectively. Then, 825 μL of the supernatant was moved into a new vial and dried in a vacuum concentrator at room temperature. Subsequently, the desiccated samples were transferred back together in 200 μL of 50% acetonitrile by sonication on an ice bath for 10 min. The remaining constituents were then subjected to centrifugation at 13,000 rpm for 15 min at 4°C, with 75 μL supernatant transferred into a new vial for LC/MS analysis. The QC samples were prepared by adding equivalent portions of the supernatant.

#### LC-TOF-MS Analysis

The UHPLC separation was carried out using a 1,290 Infinity series UHPLC System (Agilent Technologies), equipped with a UPLC BEH Amide column (2.1 * 100 mm, 1.7 μm, Waters). The analysis was performed using elution gradient as follows: 0 ∼ 0.5 min, 95% B; 0.5 ∼ 7.0 min, 95 ∼ 65% B; 7.0 ∼ 8.0 min, 65%–40% B; 8.0–9.0 min, 40% B; 9.0 ∼ 9.1 min, 40 ∼ 95% B; 9.1 ∼ 12.0 min, 95% B. The mobile phase consisted of 25 mmol/L ammonium acetate and 25 ammonia hydroxide in water (pH = 9.75) 1) and acetonitrile 2) and the column temperature was 25°C. The autosampler temperature was 4°C, and the injection volume was 1 μL (POS) and 1 μL (NEG).

During the LC–MS experiment, the TripleTOF 6,600 mass spectrometry (AB Sciex) was utilized to obtain MS–MS. The software (Analyst TF 1.7, AB Sciex) endlessly estimates the complete scan survey MS data for collecting and activating the acquirement of MS/MS spectra subject upon preselected criteria. For the individual cycles, an MS/MS at collision energy (CE) of 30 eV was used with preselected 12 ions above 100 intensity. Every cycle lasted 0.56 s. The source conditions for the ESI were established according to the following: gas 1 at 60 psi, gas 2 at 60 psi, curtain gas at 35 psi, the temperature at 600°C, declustering potential at 60 V, ion spray voltage floating at 5000 V in positive and −4000 V in the negative mode.

#### Data Preprocessing and Annotation for LC-TOF-MS Analysis

Data preprocessing was conducted in accordance with the methods described in *Data Preprocessing and Annotation for GC-TOF-MS Analysis*. The MS raw data (.wiff) files were converted to the mzXML format using the ProteoWizard and analyzed by R package XCMS (version 3.2) and preprocessed using XCMS with parameters set to peak detection, mass detection, mass detector; feature alignment: m/z tolerance 25 ppm, retention time tolerance 0.1 min. The analyses included alignment, peak deconvolution, and integration. Minfrac was established as 0.5 and the cut-offs were at 0.6. The metabolite identification was performed via an in-house MS2 database, including metlin, pubchem, and self-built database of Biotree Biotech Co., Ltd., (Shanghai, China).

### Data Analysis

The last dataset comprising of sample names, peak number information, and normalized peak areas was input into the SIMCA15.0.2 software package (Sartorius Stedim Data Analytics AB, Umea, Sweden) for multivariate analysis. The data were scaled and logarithmically transformed to reduce data variability, data noise, and large differences in variables. Principle component analysis (PCA) was conducted after the data were transformed to minimize the dimension of the data and predict the way the samples were grouped and distributed. The PCA score plot limit for identification of potential outliers in the data set was at a 95% confidence interval.

To identify metabolites that had significant deviations in the group, supervised orthogonal projections to latent structures discriminate analysis (OPLS-DA) was performed. Then, sevenfold cross-validation was performed to assess R2 and Q2. R2 specifies the way of description of the variation of a variable, while Q2 shows variable prediction. The model was permutated 200 times to evaluate the prediction and robustness of the OPLS-DA. The intercept values for R2 and Q2 were then acquired with the Q2 intercept value representative of the model robustness and reliability and the threat of overfitting. The smaller the Q2 value, the better it is. A summary of the contributions of individual variables generates the initial principal component value of the OPLS-DA analysis translating the variable importance in the projection (VIP). The metabolites with VIP >1 and *p* < 0.05 (student’s t-test) were regarded as statistically differential metabolites (SDMs). KEGG (http://www.genome.jp/kegg/) and MetaboAnalyst (http://www.metaboanalyst.ca/) databases were utilized to extend the analysis route. LC–MS-based metabolomics raw data were in Supplementary Data Sheets S2, S3.

Survival rate, retention rate, high-quality pearl rate, pearl thickness, and nacre deposition rate values among the groups were presented as mean ± SEM, with significance (*p* < 0.05) between variables detected by one-way ANOVA and subsequent Tukey’s method in IBM SPSS Statistics 19 (IBM, United States).

## Results

### Survival Rate and Pearl Trait

The survival rates recorded after the culture of pearl oysters ranged between 65.74 and 74.07%. Although the survival rates between the six groups were not significantly different, the peak survival rate was observed at EG3 (*p* > 0.05, Table 1), coupled with a peak in retention rate, which was significantly higher than EG2, EG4, EG5, and CG (Table 1, *p* < 0.05). Also, EG3 had the highest quality of pearls, with significance compared to EG1 and EG5 (Table 1, *p* < 0.05). EG3 and CG pearl thickness and nacre deposition rates were highly significant, comparing with other diets (Table 1, *p* < 0.05).

**TABLE 1:** Pearl production traits of the experimental and control groups of *P. f. martensii*.

Group	Survival rate (%)	Retention rate	High-quality pearl rate (%)	Pearl thickness (µm)	Nacre deposition rates (µm day^−1^)
CG	73.15 ± 3.09 a	0.94 ± 0.05 bc	31.11 ± 2.07 ab	231.36 ± 14.23 a	1.69 ± 0.10 a
EG1	65.74 ± 6.79 a	1.01 ± 0.02 ab	21.54 ± 5.64 b	193.84 ± 17.14 b	1.41 ± 0.13 b
EG2	73.15 ± 1.23 a	0.96 ± 0.00 b	27.69 ± 3.08 ab	184.38 ± 6.02 b	1.38 ± 0.03 b
EG3	74.07 ± 1.23 a	1.09 ± 0.04 a	35.74 ± 2.32 a	228.82 ± 8.08 a	1.67 ± 0.06 a
EG4	65.74 ± 3.09 a	0.82 ± 0.03 c	25.96 ± 3.98 ab	189.95 ± 20.89 b	1.39 ± 0.15 b
EG5	70.37 ± 3.09 a	0.89 ± 0.04 bc	20.83 ± 2.78 b	180.05 ± 10.42 b	1.31 ± 0.08 b

Within the column, means with the same letters are not significantly different (*p* > 0.05).

### General Metabolites Changes

**FIGURE 1:**
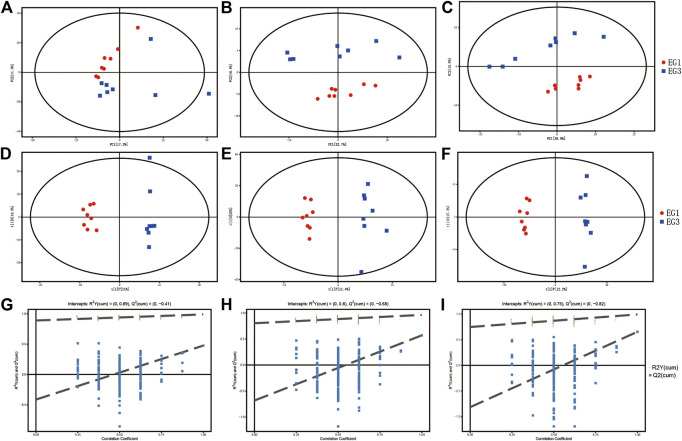
PCA model score scatter plot, OPLS-DA model, and permutation test for EG1 and EG3. The PCA model **(A–C)**, OPLS-DA model **(D–F)**, and permutation test of the OPLS-DA model **(G–I)** were derived from metabolomics profiles. A, D, and G were derived from the GC–MS; B, E, and H were derived from the LC–MS POS ion mode; and C, F, and I were derived from the LC–MS NEG ion mode.

The peak area RSD of the internal standard in the QC sample is ≤ 30%, which demonstrates the stability of the instrument data collection of GC–TOF/MS (Supplementary Table S1). In addition, the retention time and peak area of the internal standard in the sample are also stable, which demonstrates the great stability of LC–MS (Supplementary Figure S1). A total of 611 (GC–TOF/MS), 1950 (LC–MS POS), and 2,807 (LC–MS NEG) valid peaks were obtained. Gross variations in metabolic physiology were simply identified via the unsupervised PCA of the total measured analytes. The PCA and metabolic profiles of GC–MS and LC–MS are shown in Figures 1A–C. The values for R2X in the PCA among EG1 and EG3 were 0.538 (GC–MS), 0.613 (LC–MS POS), and 0.616 (LC–MS NEG). For the two groups with additional insight, we engaged OPLS-DA to investigate divergent patterns in metabolism. The OPLS-DA results are showcased in Figures 1D–F. In the OPLS-DA, the individual score scatters plot for all the samples were within the 95% Hotelling’s T-squared ellipse. The values for the R2X, R2Y, and Q2 using the OPLS-DA model of LC–MS POS among EG1 and EG3 were 0.364, 0.963, and 0.570, respectively. The values for the R2X, R2Y, and Q2 using the OPLS-DA model of GC–MS among EG1 and EG3 were 0.305, 0.988, and 0.475, respectively. The values for the R2X, R2Y, and Q2 using the OPLS-DA model of LC–MS NEG among EG1 and EG3 were 0.397, 0.992, and 0.654, respectively. The permutation test results are shown in Figures 1G–I . The permutation test was applied for confirmation to remove the transition fit of the OPLS-DA model. The test results for the R2Y and Q2 intercepts were 0.89 and −0.41 between EG1 and EG3 (GC–MS), 0.80 and −0.68 between EG1 and EG3 (LC–MS POS), and 0.75 and −0.82 between EG1 and EG3 (LC–MS NEG). Findings revealed good stability and no overfitting when using the OPLS-DA model. Therefore, it is suitable for further extension of the study.

### Metabolite Identification and Comparison

Once the metabolomics data were filtered and the noise was removed using a GC–MS-based data processing pipeline, 574 peaks remained. The metabolic library, LECO/Fiehn Rtx5, predicted that the majority of the peaks detected were endogenous metabolites. Nonetheless, certain peaks might be byproduct derivatives. A total of 275 metabolites were revealed, 100 of them by a mass spectrum similarity and with a spectral connection value (SV) of >700. A total of 2,777 and 1,916 peaks were showcased in NEG and POS after data preprocessing via LC–MS-based metabolomics, respectively. The peaks were then correlated with 419 (NEG) and 567 (POS) metabolites using the internal MS2 database.

**FIGURE 2:**
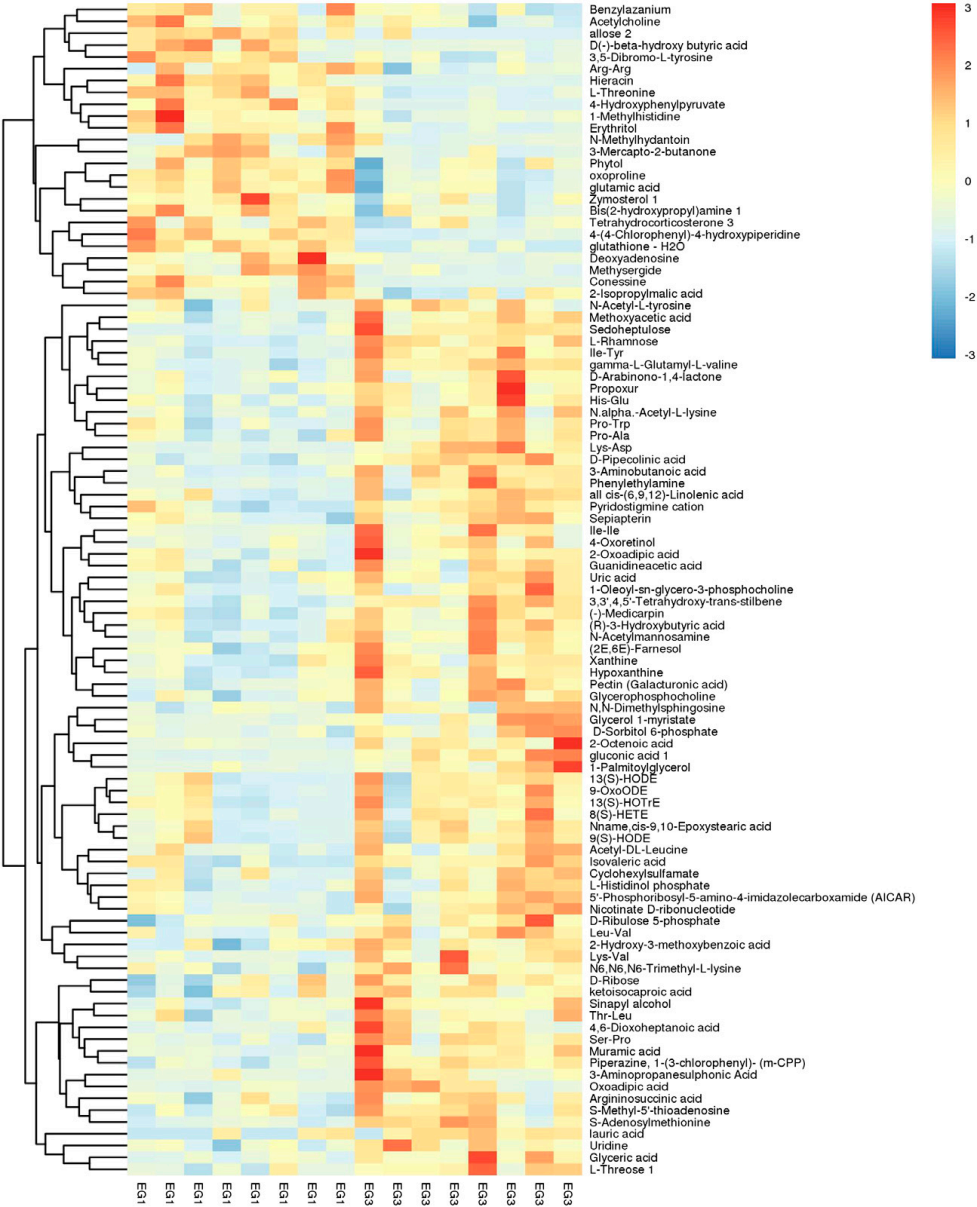
Hierarchical clustering analysis on SDMs. The relative metabolite level is depicted according to the color scale. Red and blue indicate upregulation and downregulation, respectively.

The SDMs were envisioned by hierarchical cluster analysis (Figure 2) with several metabolites being significant. A total of 135 SDMs were showcased among the EG1 and EG3 groups via LC–MS and GC–MS-based metabolomics (Figure 2; Supplementary Table S2). Comparing EG1 with EG3, EG3 had 98 SDMs with high concentrations.

### Characteristics and Functions of Key Metabolic Pathways

**FIGURE 3:**
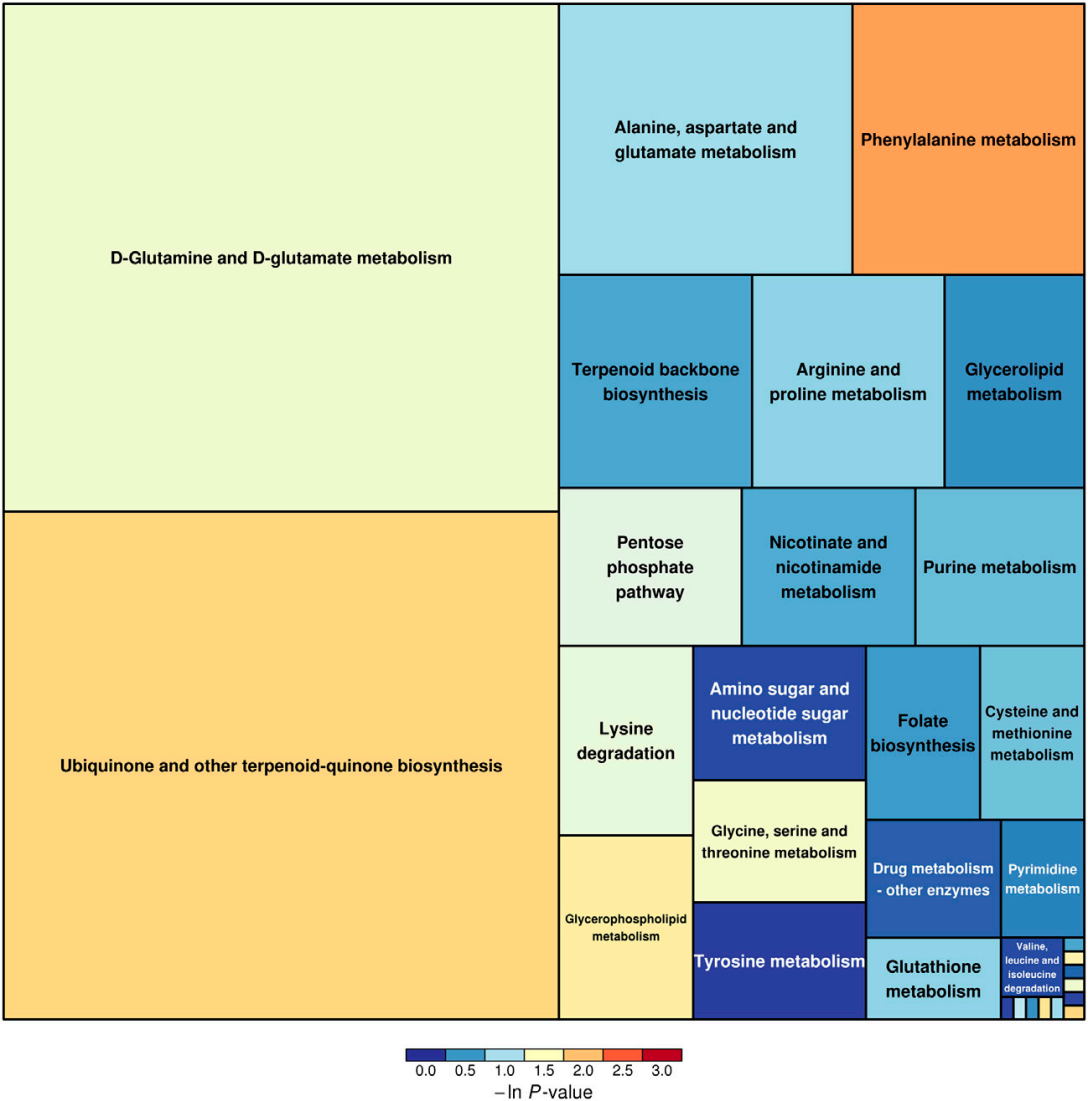
Results of metabolic pathway analysis are displayed in a rectangular tree diagram. Each block in the rectangular tree diagram represents a metabolic pathway. The sizes and colors of the block represent pathway enrichment and pathway impact, respectively. Large sizes and dark colors represent major pathway enrichment and high pathway impact values, respectively.

The SDMs, together with the acquisition from GC–MS and LC–MS, were brought into MetaboAnalyst 4.0 to evaluate the diverse possible metabolic pathways in hepatopancreases between EG1 and EG3. As shown in Figure 3, the rectangular trees demonstrated the prominent metabolic pathways associated with SDMs in hepatopancreas. Thirty-two metabolic pathways were observed among EG1 and EG3 groups (Supplementary Table S3). Regarding ln *p* value and pathway impact scores, significant metabolic pathways were observed in phenylalanine metabolism, ubiquinone, and other terpenoid-quinone biosynthesis, histidine metabolism, glycerophospholipid metabolism, D-glutamine and D-glutamate metabolism, alanine aspartate and glutamate metabolism, arginine and proline metabolism, glycerolipid metabolism, amino and nucleotide sugar metabolism, and tyrosine metabolism between EG1 and EG3 (Table 2).

**TABLE 2:** Metabolic pathways identified from the SDMs between EG1 and EG3.

Pathway	Hits	−ln (p)	Impact	Hits cpd
Phenylalanine metabolism	2	2.166	0.222	Phenylethylamine cpd:C05332
				2-Phenylacetamide cpd:C02505
Ubiquinone and other terpenoid-quinone biosynthesis	1	1.878	1.000	4-Hydroxyphenylpyruvic acid cpd:C01179
Histidine metabolism	2	1.765	0.000	L-Glutamic acid cpd:C00025
				1-Methylhistidine cpd:C01152
Glycerophospholipid metabolism	3	1.674	0.087	LysoPC (18:1 (9Z)) cpd:C04230
				Acetylcholine cpd:C01996
				Glycerophosphocholine cpd:C00670
D-Glutamine and D-glutamate metabolism	1	1.420	1.000	L-Glutamic acid cpd:C00025
Pentose phosphate pathway	2	1.301	0.101	D-Ribose cpd:C00121
				D-Ribulose 5-phosphate cpd:C00199
Alanine, aspartate, and glutamate metabolism	2	0.985	0.282	Argininosuccinic acid cpd:C03406
				L-Glutamic acid cpd:C00025
Arginine and proline metabolism	3	0.890	0.145	Argininosuccinic acid cpd:C03406
				L-Glutamic acid cpd:C00025
				Guanidoacetic acid cpd:C00581
Purine metabolism	4	0.734	0.094	Xanthine cpd:C00385
				Deoxyadenosine cpd:C00559
				Hypoxanthine cpd:C00262
				Uric acid cpd:C00366
Glycerolipid metabolism	1	0.458	0.105	Glyceric acid cpd:C00258
Amino sugar and nucleotide sugar metabolism	1	0.134	0.082	N-Acetylmannosamine cpd:C00645
Tyrosine metabolism	1	0.088	0.071	4-Hydroxyphenylpyruvic acid cpd:C01179

Hits is the number of significantly differential metabolites in one pathway. *p* value is calculated from the pathway enrichment analysis. Impact represents impact value in the pathway topology analysis.

## Discussion

Increasing the yield and quality of cultured pearls is important for pearl production, and it is also a challenging venture in research and development. Traditionally, pearl oysters are suspended in the sea, vulnerable to changing environments (e.g., temperature, pollution, biofouling, pathogens, and phytoplankton biomass) (Latchere et al., 2017; Li et al., 2018; Adzigbli et al., 2020). Land-based facilities for pearl culture not only provide constant environment mobilization of contaminants by natural disasters and pollution, but also offer better nutritional access to enhance biomineralization capacity of host oysters and improve pearl quality and yield (Yang et al., 2017a; Yang et al., 2017b). Unfortunately, dietary demand is on the high side, and the availability of formulated diets is quite limited. As typical filter-feeding species, the pearl oyster *P. f. martensii* may take up water-suspended particles as food sources, like microzooplankton and algae, bacteria, and organic debris (Wang et al., 2016). Conversely, microalgal culturing is labor intensive and difficult to control in large-scale production. Hence, dietary requirements for commercial farms might not be catered for by this process. Since 2015, our lab has been developing formulated diets of *P. f. martensii* (Yang et al., 2017a; Yang et al., 2017b; Yang et al., 2018a; Yang et al., 2018b; Yang et al., 2019a; Yang et al., 2019b) and proven that land-based cultures can boost survival and retention rates of host pearl oysters. In the present research, land-based cultured EG3 showed heightened pearl production (survival rate, retention rate, high-quality pearl rate, pearl thickness, and nacre deposition rates). Our findings indicate that the improvement of the culture environment and the reduction of the peripheral disturbances could reduce the mortality of host pearl oysters. Besides, a diet with VD3 optimum levels also increases pearl production traits.

Pearls result from pearl oyster metabolism, comprising of CaCO_3_ (91.5%) with fragments of organic substances (3.83%), residual substances (0.01%), and water (3.97%) (Southgate and Lucas, 2008). VD3 can regulate calcium metabolism, improve the absorption of calcium, increase the utilization of calcium, and maintain calcification. However, the effect of VD3 on mineralization and the deposition of calcium and phosphorus is concentration-dependent (i.e., within an appropriate level). Increased VD3 content can promote mineralization and calcium and phosphorus deposition, but too high or too low content can cause abnormal mineralization. Previous results have shown that the appropriate level of VD3 addition promotes the absorption of calcium by laying hens and enhances their production performance (Kang et al., 2018). The lack of VD3 in *Penaeus vannamei* shows an incomplete shrimp shell mineralization (He et al., 1992; Rai and Reddy, 2004). The appropriate level of VD3 feed strengthens the mineralization ability of juvenile Japanese seabass skeleton (Zhang et al., 2016). The proper addition of VD3 in the feed also promotes the deposition of minerals in the abalone shells (Zhou and Mai, 2004). With this study, the pearl thickness and nacre deposition rates of pearls in the EG3 were highly significant than those of VD3 level-added group. This result suggests that an appropriate amount of VD3 in the feed can promote pearlescent deposition, but insufficient or excessive addition of VD3 inhibits minerals in the accumulation in pearls, which is consistent with the aforementioned studies (Zhou and Mai, 2004; Zhang et al., 2016).

Effective strategies in adapting to different dietary VD3 levels must be clarified, and the mechanism underlying this phenomenon should be explored to develop heightened nutritional supplies and attain optimal pearl production traits. Metabolomics is a useful tool to obtain a whole-organism overview of affected pathways, and SDMs indicate characteristics of nutritional or therapeutic intervention in organisms (Ma et al., 2017). To determine the metabolomic responses of pearl oyster given a low-VD3 diet, LC–MS and GC–MS were performed. To explore the differences in the hepatopancreases of the host pearl oysters, a comparison between EG1 and EG3 metabolites was conducted. Significant differences were found in the majority of the metabolites among the two groups, such as ubiquinone and other terpenoid-quinone biosynthesis; D-glutamine and D-glutamate metabolism; phenylalanine metabolism; alanine, aspartate, and glutamate metabolism; glycerophospholipid metabolism; pentose phosphate pathway; and glycine, serine, and threonine metabolism.

Biomineralization is the process by which living forms influence the precipitation of mineral materials not based on chemistry but according to effective biological facilitation and control, like a shell, pearl, bone, and eggshell (Cui, 2007). Du et al. (2017) reported *P. f. martensii* shell as a prototype for nacre formation, in which chitin is the organic matrix core for the formation. Von Willebrand factor A domain-containing protein (VWAP) with chitin-binding domains connects to chitin and interrelates with other VWAPs and fibronectins, establishing the matrix networks. The ECM-related proteins represent focal elements participating in shell formation. ECM is a complex mixture of functional and structural macromolecules, like fibrous proteins (e.g., elastin, collagen, fibronectin, and lammin) and glycosaminoglycans (GAGs) (Hao et al., 2019). GAGs with substantial levels of negative sulfates can augment calcium concentration and cause supersaturation, which is essential for nucleation of the structured carboxylate domains (Hao et al., 2018b). During amino and nucleotide sugar metabolisms, the N-acetylmannosamine levels of EG3 were higher than those of EG1. N-Acetylmannosamine is converted to N-acetylneuramine acid, a terminal component in many glycoproteins and glycolipids (Shi et al., 2015). This could partly explain the poorer pearl production traits in EG1 compared with those in EG3.

Amino acids are the main elevated metabolites discovered in high pearl production traits group (EG3), including phenylalanine, N-acetyl-L-tyrosine, and acetyl-DL-Leucine. The increased phenylalanine can trigger the calcium receptor and subdue the emission of the parathyroid hormone (Lee et al., 2007), subduing the discharge of calcium from bone into blood (Juppner et al., 1991). In addition, phenylalanine is vital for the binding of BMP (Kirsch et al., 2000; Groppe et al., 2002), and Pm-BMP7 functions crucially in prismatic and nacre layer formation of the shell (Yan et al., 2014). Small leucine-rich protein is a mineralized tissue-specific protein (Sugars et al., 2013) involved in regulating collagen fibrillogenesis, matrix mineralization, and osteoblast differentiation (Mochida et al., 2011). Tyrosine is associated with tyrosine kinase and phosphatase activities (Pallu et al., 2012). It is involved in the transduction by controlling dephosphorylation and tyrosine protein phosphorylation (Alonso and Pulido, 2016) and is vital for osteoclast’s function and formation (Schmidt et al., 1996). Tyrosine is a substrate for tyrosinase, which catalyzes dopamine and tyrosine oxidation and has extensive involvement in the shell maturation. Thus, it could be important for the assembling and maturation of prismatic and nacreous shell matrices (Du et al., 2017). This study presented elevated levels of related-metabolites (phenylalanine, Leu-Val, Thr-Leu, tyrosine, and acetyl-DL-Leucine), which might result in high pearl production traits in EG3. Xu et al. (2017) reported that the elevation of pyroglutamic acid (oxoproline) may indicate that excessive oxygen free radicals are produced, resulting in impaired chondrocytes and collagen. In the present study, oxoproline had higher content in EG1 than in EG3 (FC = 1.306), which explained that pearl oyster in EG3 possessed high biomineralization activity.

Lipids are also mined from oyster nacre and can play an essential function in biomineralization and fossilization (Farre and Dauphin, 2009). The interruption of lipid rafts influences actin ring formation and leads to apoptosis in osteoclasts. Therefore, glycosphingolipids are vital for osteoclastogenesis through lipid rafts (Fukumoto et al., 2006). Osteoclast and osteoblast in the bone can be influenced by glycerophospholipids (Zhu et al., 2016). Hyaluronic acid can also promote cell aggregation to form a colloidal matrix and regulate the concentration and transport of Ca^2+^ (Yu et al., 2005). Phosphatidyl choline can form complexes with hyaluronic acids (Xu et al., 2017). In the glycerophospholipid metabolism, the sn-glycero-3-phosphocholine and 1-acyl-sn-glycero-3-phosphocholine contents in EG1 were lower than those in EG3, and the acetylcholine contents in EG1 were higher than those in EG3. This finding is similar to previous results where glycerophospholipids metabolism is vital in regulating bone metabolism abnormality (Zhu et al., 2016). The disordered purine metabolism causes reduced osteoclastogenesis and an inherent deficiency of osteoblast function with successive low bone formation (Sauer et al., 2009), which influences bone biomineralization. Decreased xanthin, deoxyadenosine, hypoxanthine and uric acid were involved in purine metabolism in EG1. This explained that pearl oyster in EG1 possessed low biomineralization activity.

## Conclusion

Dietary VD3 levels in formulated diets significantly affect the retention rate, high-quality pearl rate, pearl thickness, and nacre deposition rates. The effects of low doses of VD3 on *P. f. martensii* pearl production traits were assessed using the GC–MS and LC–MS metabolomics. A total of 135 SDMs were revealed and observed to be involved in 32 pathways. These analyses are a step further in the main metabolic pathways to reveal the ability of pearl oyster to possess diverse metabolic capabilities. These metabolic capabilities involve phenylalanine metabolism, histidine metabolism, glycerophospholipid metabolism, alanine aspartate and glutamate metabolism, arginine and proline metabolism, glycerolipid metabolism, amino sugar and nucleotide sugar metabolism, and tyrosine metabolism. Results from this study will provide a theoretical foundation for understanding the impacts of VD3 on pearl production traits of pearl oyster and strengthen impending prospects and application of VD3 in pearl oyster aquaculture.

## Data Availability

The original contributions presented in the study are included in the article/Supplementary Material; further inquiries can be directed to the corresponding authors.
