# Supplementary material for: Integrated GC–MS- and LC–MS-Based Untargeted Metabolomics Studies of the Effect of Vitamin D3 on Pearl Production Traits in Pearl Oyster Pinctada fucata martensii
Source: Front Mol Biosci. 2021 Mar 5;8:614404. doi: 10.3389/fmolb.2021.614404 (PMC7973263; doi:10.3389/fmolb.2021.614404)
Supplement: Supplementary file 1 [file table1.docx]

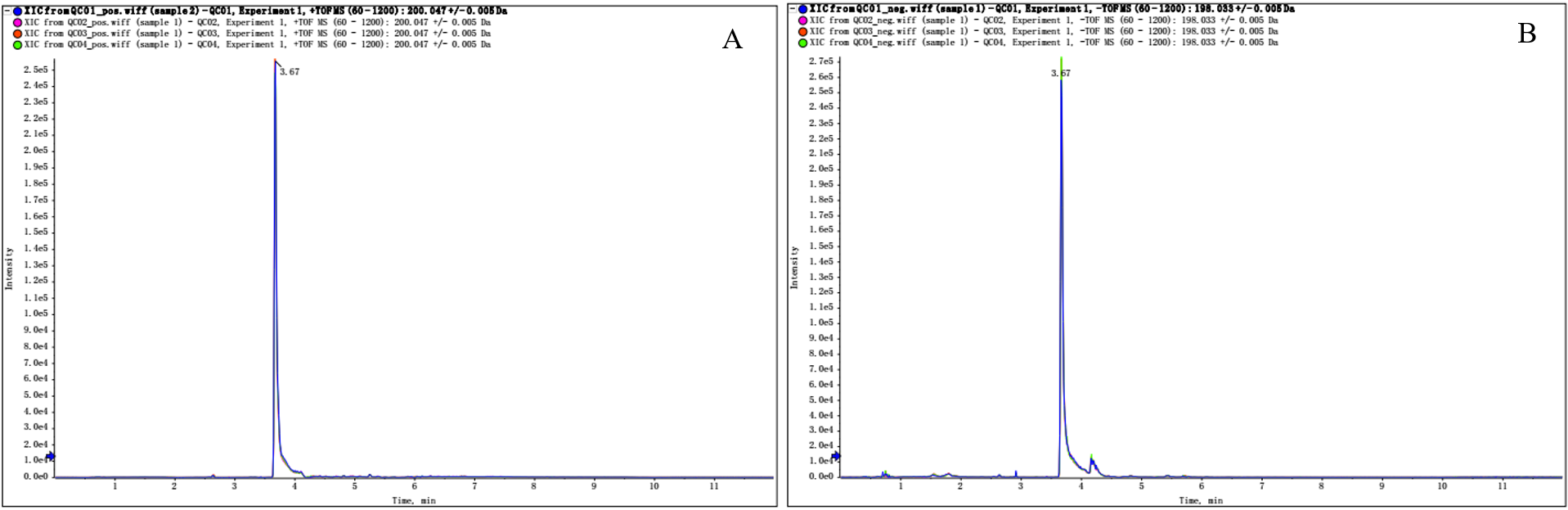


Supplementary Fig. 1 The retention time and peak area of the internal standard in the sample (A:POS, B:NEG)
