# Supplementary material for: Integrated GC–MS- and LC–MS-Based Untargeted Metabolomics Studies of the Effect of Vitamin D3 on Pearl Production Traits in Pearl Oyster Pinctada fucata martensii
Source: Front Mol Biosci. 2021 Mar 5;8:614404. doi: 10.3389/fmolb.2021.614404 (PMC7973263; doi:10.3389/fmolb.2021.614404)
Supplement: Supplementary file 2 [file table2.docx]

Supplementary Table 1 The peak area of the internal standard in the QC sample

| Sample | Area | Sample | Area |
| --- | --- | --- | --- |
| QC1_1 | 358236 | QC3_1 | 421537 |
| QC1_2 | 361199 | QC4_1 | 518816 |
| QC2_1 | 361733 | - | - |
| Average | **404304** | **RSD** | **17.14%** |
