# Supplementary material for: Integrated GC–MS- and LC–MS-Based Untargeted Metabolomics Studies of the Effect of Vitamin D3 on Pearl Production Traits in Pearl Oyster Pinctada fucata martensii
Source: Front Mol Biosci. 2021 Mar 5;8:614404. doi: 10.3389/fmolb.2021.614404 (PMC7973263; doi:10.3389/fmolb.2021.614404)
Supplement: Supplementary file 3 [file table3.docx]

Supplementary Table 2 Identification of SDMs in the hepatopancreas between EG1 and EG3

| compound name | score | rt | mz | platform | VIP | P-value | FC |
| --- | --- | --- | --- | --- | --- | --- | --- |
| 1-Palmitoylglycerol | 0.730 | 136.094 | 313.276 | POS | 2.001 | 0.023 | 0.179 |
| Enterostatin human | 0.935 | 34.807 | 495.275 | NEG | 2.370 | 0.008 | 0.195 |
| Phenylethylamine | 0.864 | 164.101 | 122.097 | POS | 2.101 | 0.004 | 0.200 |
| Mevalonic acid | 0.992 | 29.215 | 295.135 | NEG | 2.645 | 0.001 | 0.241 |
| gluconic acid 1 | 0.839 | 1141.056 | 333.000 | GC | 2.467 | 0.003 | 0.246 |
| 3-Aminopropanesulphonic Acid | 0.999 | 249.435 | 138.023 | NEG | 1.834 | 0.049 | 0.308 |
| Aceclidine | 0.913 | 336.816 | 170.119 | POS | 2.606 | 0.000 | 0.354 |
| Pirenzepine | 0.958 | 313.680 | 332.144 | NEG | 2.336 | 0.017 | 0.367 |
| Lys-Asp | 0.997 | 330.625 | 226.120 | POS | 2.472 | 0.002 | 0.379 |
| lauric acid | 0.505 | 927.978 | 117.000 | GC | 1.326 | 0.027 | 0.384 |
| Ile-Ile | 0.999 | 365.375 | 283.142 | POS | 2.032 | 0.037 | 0.388 |
| Sedoheptulose | 0.947 | 27.887 | 231.047 | NEG | 2.531 | 0.002 | 0.389 |
| Glycerol 1-myristate | 0.930 | 30.771 | 285.245 | POS | 1.294 | 0.040 | 0.390 |
| Adipic acid | 0.679 | 404.271 | 127.039 | NEG | 1.994 | 0.042 | 0.397 |
| L-Rhamnose | 0.719 | 366.668 | 206.103 | POS | 2.550 | 0.000 | 0.398 |
| gamma-L-Glutamyl-L-valine | 0.971 | 352.145 | 211.106 | POS | 2.470 | 0.000 | 0.398 |
| Pravastatin | 0.702 | 157.437 | 461.185 | NEG | 1.785 | 0.035 | 0.421 |
| Altretamine | 0.977 | 484.935 | 175.145 | POS | 2.743 | 0.000 | 0.430 |
| 2'-Deoxyuridine | 0.903 | 112.510 | 227.067 | NEG | 1.371 | 0.044 | 0.435 |
| 3,3',4,5'-Tetrahydroxy-trans-stilbene | 0.706 | 153.425 | 487.128 | NEG | 2.355 | 0.000 | 0.438 |
| D-Pipecolinic acid | 0.981 | 334.348 | 171.114 | POS | 2.354 | 0.000 | 0.443 |
| 3-Aminobutanoic acid | 0.972 | 151.508 | 229.120 | POS | 2.306 | 0.001 | 0.449 |
| 1-Oleoyl-sn-glycero-3-phosphocholine | 0.848 | 180.194 | 566.322 | POS | 1.481 | 0.039 | 0.452 |
| Ile-Tyr | 0.995 | 376.609 | 294.157 | POS | 2.532 | 0.001 | 0.452 |
| Nname,cis-9,10-Epoxystearic acid | 0.984 | 62.809 | 297.243 | NEG | 1.316 | 0.025 | 0.454 |
| 2-Octenoic acid | 0.677 | 400.879 | 209.049 | POS | 1.924 | 0.022 | 0.463 |
| Muramic acid | 0.997 | 365.560 | 501.190 | NEG | 2.240 | 0.015 | 0.477 |
| Piperazine, 1-(3-chlorophenyl)- (m-CPP) | 0.757 | 304.844 | 214.109 | POS | 2.342 | 0.000 | 0.495 |
| S-Methyl-5'-thioadenosine | 1.000 | 97.606 | 298.100 | POS | 1.672 | 0.019 | 0.498 |
| 4,6-Dioxoheptanoic acid | 0.702 | 279.109 | 222.078 | POS | 1.817 | 0.046 | 0.499 |
| S-Adenosylmethionine | 0.977 | 444.469 | 399.147 | POS | 2.256 | 0.002 | 0.513 |
| N,N-Dimethylsphingosine | 0.775 | 157.023 | 388.335 | POS | 1.545 | 0.009 | 0.523 |
| Lys-Val | 0.992 | 398.019 | 228.172 | POS | 2.106 | 0.011 | 0.531 |
| Oxoadipic acid | 0.886 | 121.572 | 219.048 | NEG | 1.710 | 0.029 | 0.550 |
| 9-OxoODE | 0.884 | 63.020 | 293.210 | NEG | 1.234 | 0.040 | 0.552 |
| Glyceric acid | 0.690 | 120.178 | 87.009 | NEG | 2.154 | 0.015 | 0.557 |
| 9(S)-HODE | 0.936 | 63.552 | 295.226 | NEG | 1.238 | 0.043 | 0.559 |
| D-Sorbitol 6-phosphate | 0.717 | 191.941 | 523.291 | NEG | 1.864 | 0.010 | 0.571 |
| all cis-(6,9,12)-Linolenic acid | 0.860 | 45.789 | 279.233 | POS | 1.587 | 0.017 | 0.574 |
| 8(S)-HETE | 0.765 | 45.827 | 319.227 | NEG | 1.614 | 0.027 | 0.582 |
| Ethylmalonic acid | 0.754 | 375.023 | 282.121 | POS | 1.844 | 0.008 | 0.587 |
| Nicotinate D-ribonucleotide | 0.600 | 415.874 | 351.055 | NEG | 1.288 | 0.032 | 0.595 |
| Ketanserin | 0.946 | 39.827 | 394.167 | NEG | 2.414 | 0.002 | 0.596 |
| Trimethobenzamide | 0.990 | 368.090 | 449.228 | POS | 1.260 | 0.043 | 0.601 |
| 4-Oxoretinol | 0.645 | 45.590 | 301.219 | POS | 1.965 | 0.023 | 0.601 |
| 5'-Phosphoribosyl-5-amino-4-imidazolecarboxamide (AICAR) | 0.901 | 416.277 | 338.070 | NEG | 1.935 | 0.006 | 0.606 |
| Sepiapterin | 0.671 | 256.688 | 296.096 | NEG | 1.837 | 0.011 | 0.612 |
| 5'-Deoxyadenosine | 1.000 | 158.373 | 274.089 | POS | 1.977 | 0.015 | 0.615 |
| 13(S)-HODE | 0.875 | 46.418 | 295.227 | NEG | 1.294 | 0.048 | 0.616 |
| Zearalenone | 0.963 | 314.223 | 377.154 | NEG | 1.812 | 0.013 | 0.621 |
| N.alpha.-Acetyl-L-lysine | 0.964 | 436.317 | 249.147 | POS | 2.005 | 0.009 | 0.621 |
| Ser-Pro | 0.681 | 374.705 | 266.107 | POS | 2.195 | 0.004 | 0.622 |
| Astemizole | 0.994 | 36.088 | 457.250 | NEG | 1.379 | 0.049 | 0.622 |
| D-Ribose | 0.996 | 72.654 | 209.067 | NEG | 1.638 | 0.037 | 0.625 |
| ketoisocaproic acid | 0.899 | 44.424 | 129.055 | NEG | 1.436 | 0.047 | 0.630 |
| 13(S)-HOTrE | 0.620 | 46.445 | 293.211 | NEG | 1.535 | 0.033 | 0.632 |
| Propoxur | 0.884 | 297.345 | 254.071 | POS | 1.861 | 0.035 | 0.632 |
| Sinapyl alcohol | 0.885 | 188.841 | 249.056 | POS | 2.060 | 0.014 | 0.636 |
| Acetyl-DL-Leucine | 0.998 | 45.967 | 215.142 | POS | 1.514 | 0.044 | 0.642 |
| 5-Methylcytidine | 1.000 | 396.685 | 275.137 | POS | 1.656 | 0.042 | 0.645 |
| L-Threose 1 | 0.666 | 764.592 | 201.000 | GC | 1.948 | 0.008 | 0.646 |
| RU-0211 | 0.987 | 208.150 | 391.221 | POS | 1.599 | 0.031 | 0.647 |
| Xanthine | 0.982 | 201.875 | 153.041 | POS | 1.845 | 0.015 | 0.655 |
| Cyclohexylamine | 0.832 | 363.130 | 160.135 | POS | 1.649 | 0.016 | 0.657 |
| Hypoxanthine | 0.998 | 158.376 | 137.048 | POS | 2.245 | 0.003 | 0.657 |
| Evoxine | 0.924 | 391.596 | 330.134 | POS | 2.067 | 0.004 | 0.661 |
| Uridine | 0.996 | 153.546 | 245.079 | POS | 1.947 | 0.008 | 0.674 |
| Uric acid | 0.858 | 177.001 | 167.020 | NEG | 1.976 | 0.006 | 0.674 |
| Fluoxetine | 0.999 | 440.319 | 292.136 | POS | 1.904 | 0.017 | 0.681 |
| (2E,6E)-Farnesol | 0.754 | 62.584 | 337.240 | POS | 1.629 | 0.042 | 0.682 |
| Argininosuccinic acid | 0.985 | 440.256 | 291.133 | POS | 1.715 | 0.032 | 0.692 |
| Dantrolene | 0.907 | 184.495 | 295.051 | NEG | 2.108 | 0.002 | 0.707 |
| Terbutaline | 0.972 | 417.615 | 289.153 | POS | 2.010 | 0.005 | 0.712 |
| D-Arabinono-1,4-lactone | 0.952 | 148.686 | 166.073 | POS | 1.764 | 0.048 | 0.717 |
| Cyclohexylsulfamate | 0.838 | 84.504 | 238.074 | NEG | 1.518 | 0.042 | 0.719 |
| His-Glu | 0.641 | 348.165 | 267.112 | POS | 2.003 | 0.011 | 0.725 |
| N-Acetyl-L-tyrosine | 0.890 | 322.370 | 246.075 | POS | 1.640 | 0.032 | 0.727 |
| Tetrahydrobiopterin | 0.928 | 259.094 | 240.105 | NEG | 1.594 | 0.045 | 0.729 |
| N6,N6,N6-Trimethyl-L-lysine | 0.999 | 505.227 | 189.162 | POS | 1.827 | 0.011 | 0.732 |
| Quetiapine | 0.980 | 365.634 | 348.150 | POS | 1.477 | 0.046 | 0.733 |
| Pyridostigmine cation | 0.946 | 334.969 | 199.128 | POS | 2.031 | 0.006 | 0.739 |
| Desipramine | 0.985 | 281.722 | 231.162 | POS | 2.108 | 0.002 | 0.740 |
| Leu-Val | 0.995 | 281.728 | 230.161 | POS | 2.121 | 0.003 | 0.740 |
| Thr-Leu | 0.691 | 98.604 | 255.136 | POS | 1.628 | 0.049 | 0.744 |
| N-Acetylmannosamine | 0.856 | 262.563 | 204.088 | POS | 2.054 | 0.008 | 0.745 |
| Methoxyacetic acid | 1.000 | 379.089 | 132.067 | POS | 2.175 | 0.004 | 0.748 |
| L-Histidinol phosphate | 0.713 | 432.056 | 263.089 | POS | 1.648 | 0.030 | 0.750 |
| Pectin (Galacturonic acid) | 1.000 | 365.292 | 255.067 | POS | 2.113 | 0.009 | 0.755 |
| Isovaleric acid | 0.803 | 310.650 | 139.017 | NEG | 2.003 | 0.007 | 0.756 |
| Pro-Ala | 0.980 | 404.329 | 247.131 | POS | 2.018 | 0.007 | 0.763 |
| (R)-3-Hydroxybutyric acid | 0.899 | 243.173 | 168.067 | POS | 1.913 | 0.015 | 0.766 |
| (-)-Medicarpin | 0.992 | 203.642 | 270.094 | POS | 1.965 | 0.012 | 0.770 |
| Glycerophosphocholine | 0.991 | 415.865 | 258.111 | POS | 1.849 | 0.016 | 0.781 |
| 2-Oxoadipic acid | 0.972 | 333.414 | 159.029 | NEG | 1.792 | 0.042 | 0.785 |
| 2-Hydroxy-3-methoxybenzoic acid | 0.766 | 365.390 | 337.090 | POS | 1.477 | 0.045 | 0.804 |
| D-Ribulose 5-phosphate | 0.805 | 272.679 | 211.000 | NEG | 1.606 | 0.018 | 0.804 |
| Pro-Trp | 0.994 | 389.440 | 266.125 | POS | 1.796 | 0.029 | 0.809 |
| Guanidineacetic acid | 0.714 | 35.151 | 178.079 | POS | 1.684 | 0.039 | 0.812 |
| Phytol | 0.284 | 1240.302 | 71.000 | GC | 1.712 | 0.022 | 1.237 |
| Pseudoephedrine | 0.724 | 294.719 | 226.145 | POS | 1.416 | 0.037 | 1.269 |
| oxoproline | 0.937 | 836.682 | 156.000 | GC | 2.327 | 0.000 | 1.306 |
| Arg-Arg | 0.716 | 220.347 | 372.240 | POS | 1.366 | 0.036 | 1.355 |
| Acetylcholine | 0.999 | 48.718 | 184.077 | POS | 1.654 | 0.018 | 1.357 |
| glutamic acid | 0.927 | 902.406 | 246.000 | GC | 2.109 | 0.002 | 1.400 |
| 2-Phenylacetamide | 0.990 | 40.832 | 177.103 | POS | 1.124 | 0.046 | 1.428 |
| 2-Isopropylmalic acid | 0.796 | 89.493 | 194.105 | POS | 1.420 | 0.037 | 1.483 |
| Zymosterol 1 | 0.832 | 1746.000 | 69.000 | GC | 1.729 | 0.039 | 1.526 |
| Tetrahydrocorticosterone 3 | 0.565 | 1639.806 | 103.000 | GC | 1.766 | 0.027 | 1.532 |
| Floxuridine | 0.611 | 418.525 | 246.061 | NEG | 2.274 | 0.000 | 1.569 |
| 1-Indanone | 0.606 | 49.203 | 303.076 | POS | 1.592 | 0.025 | 1.577 |
| Bis(2-hydroxypropyl)amine 1 | 0.289 | 800.850 | 174.000 | GC | 1.820 | 0.010 | 1.675 |
| 3,5-Dibromo-L-tyrosine | 0.833 | 36.198 | 352.909 | NEG | 1.679 | 0.009 | 1.683 |
| 3-Mercapto-2-butanone | 0.869 | 298.332 | 165.060 | POS | 1.745 | 0.016 | 1.923 |
| Benzylazanium | 0.904 | 81.996 | 168.103 | POS | 1.379 | 0.030 | 1.934 |
| 1-Methylhistidine | 0.901 | 147.364 | 170.093 | POS | 1.665 | 0.029 | 2.035 |
| Galangin | 0.999 | 39.676 | 269.044 | NEG | 2.171 | 0.001 | 2.103 |
| L-Threonine | 0.998 | 357.261 | 178.071 | NEG | 2.319 | 0.000 | 2.219 |
| Pinosylvin | 0.791 | 70.479 | 233.058 | NEG | 1.861 | 0.003 | 2.326 |
| Methysergide | 0.845 | 208.120 | 371.240 | POS | 2.095 | 0.006 | 2.360 |
| Clozapine | 0.989 | 292.328 | 390.139 | POS | 1.881 | 0.011 | 2.429 |
| Streptozocin | 0.626 | 106.981 | 264.089 | NEG | 1.802 | 0.002 | 2.438 |
| N-Methylhydantoin | 0.985 | 326.928 | 132.077 | POS | 1.326 | 0.042 | 2.452 |
| Erythritol | 0.779 | 375.223 | 267.111 | POS | 2.211 | 0.004 | 2.645 |
| Ammelide | 0.856 | 98.186 | 144.056 | NEG | 2.353 | 0.003 | 2.839 |
| Deoxyadenosine | 1.000 | 135.660 | 252.112 | POS | 1.942 | 0.032 | 2.906 |
| 4-(4-Chlorophenyl)-4-hydroxypiperidine | 0.675 | 287.443 | 194.077 | POS | 2.663 | 0.000 | 3.279 |
| Dioscin | 0.995 | 188.436 | 905.450 | NEG | 2.638 | 0.000 | 3.588 |
| D(-)-beta-hydroxy butyric acid | 0.964 | 221.655 | 103.040 | NEG | 1.946 | 0.011 | 3.681 |
| Guanfacine | 0.692 | 39.919 | 263.049 | POS | 2.480 | 0.000 | 4.910 |
| Hieracin | 0.836 | 20.479 | 318.058 | NEG | 2.622 | 0.001 | 6.702 |
| 4-Hydroxyphenylpyruvate | 0.675 | 121.433 | 163.043 | POS | 2.495 | 0.001 | 6.743 |
| glutathione - H2O | 0.337 | 1457.586 | 210.000 | GC | 2.183 | 0.000 | 7.051 |
| allose 2 | 0.735 | 1091.364 | 363.000 | GC | 2.154 | 0.001 | 7.265 |
| Estradiol Cypionate | 0.852 | 229.383 | 395.265 | NEG | 2.547 | 0.004 | 7.534 |
| Conessine | 0.983 | 156.363 | 357.325 | POS | 2.634 | 0.002 | 17.547 |
