# Supplementary material for: Integrated GC–MS- and LC–MS-Based Untargeted Metabolomics Studies of the Effect of Vitamin D3 on Pearl Production Traits in Pearl Oyster Pinctada fucata martensii
Source: Front Mol Biosci. 2021 Mar 5;8:614404. doi: 10.3389/fmolb.2021.614404 (PMC7973263; doi:10.3389/fmolb.2021.614404)
Supplement: Supplementary file 4 [file table4.docx]

Supplementary Table 3 Metabolic pathways identified on the SDMs from the hepatopancreas between EG1 and EG3

| Pathway | Hits | -ln(p) | Impact | Hits Cpd |
| --- | --- | --- | --- | --- |
| Phenylalanine metabolism | 2 | 2.166 | 0.222 | Phenylethylamine cpd:C05332; 2-Phenylacetamide cpd:C02505 |
| Valine, leucine and isoleucine biosynthesis | 2 | 1.885 | 0.000 | L-Threonine cpd:C00188; 4-Methyl-2-oxopentanoate cpd:C00233 |
| Ubiquinone and other terpenoid-quinone biosynthesis | 1 | 1.878 | 1.000 | 4-Hydroxyphenylpyruvic acid cpd:C01179 |
| Histidine metabolism | 2 | 1.765 | 0.000 | L-Glutamic acid cpd:C00025; 1-Methylhistidine cpd:C01152 |
| Glycerophospholipid metabolism | 3 | 1.674 | 0.087 | LysoPC (18:1(9Z)) cpd:C04230; Acetylcholine cpd:C01996; Glycerophosphocholine cpd:C00670 |
| Phenylalanine, tyrosine and tryptophan biosynthesis | 1 | 1.617 | 0.000 | 4-Hydroxyphenylpyruvic acid cpd:C01179 |
| Glycine, serine and threonine metabolism | 3 | 1.468 | 0.074 | Glyceric acid cpd:C00258; Guanidoacetic acid cpd:C00581; L-Threonine cpd:C00188 |
| Synthesis and degradation of ketone bodies | 1 | 1.420 | 0.000 | (R)-3-Hydroxybutyric acid cpd:C01089 |
| D-Glutamine and D-glutamate metabolism | 1 | 1.420 | 1.000 | L-Glutamic acid cpd:C00025 |
| Lysine degradation | 2 | 1.379 | 0.090 | N6,N6,N6-Trimethyl-L-lysine cpd:C03793; Oxoadipic acid cpd:C00322 |
| Pentose phosphate pathway | 2 | 1.301 | 0.101 | D-Ribose cpd:C00121; D-Ribulose 5-phosphate cpd:C00199 |
| Butanoate metabolism | 2 | 1.099 | 0.000 | (R)-3-Hydroxybutyric acid cpd:C01089; L-Glutamic acid cpd:C00025 |
| Alanine, aspartate and glutamate metabolism | 2 | 0.985 | 0.282 | Argininosuccinic acid cpd:C03406; L-Glutamic acid cpd:C00025 |
| Nitrogen metabolism | 1 | 0.934 | 0.000 | L-Glutamic acid cpd:C00025 |
| Arginine and proline metabolism | 3 | 0.890 | 0.145 | Argininosuccinic acid cpd:C03406; L-Glutamic acid cpd:C00025; Guanidoacetic acid cpd:C00581 |
| Glutathione metabolism | 2 | 0.886 | 0.038 | L-Glutamic acid cpd:C00025; Pyroglutamic acid cpd:C01879 |
| Cysteine and methionine metabolism | 2 | 0.758 | 0.063 | 5'-Methylthioadenosine cpd:C00170; S-Adenosylmethionine cpd:C00019 |
| Purine metabolism | 4 | 0.734 | 0.094 | Xanthine cpd:C00385; Deoxyadenosine cpd:C00559; Hypoxanthine cpd:C00262; Uric acid cpd:C00366 |
| Nicotinate and nicotinamide metabolism | 1 | 0.615 | 0.096 | Nicotinic acid mononucleotide cpd:C01185 |
| Pentose and glucuronate interconversions | 1 | 0.570 | 0.000 | D-Ribulose 5-phosphate cpd:C00199 |
| Terpenoid backbone biosynthesis | 1 | 0.570 | 0.145 | Mevalonic acid cpd:C00418 |
| Folate biosynthesis | 1 | 0.529 | 0.070 | Tetrahydrobiopterin cpd:C00272 |
| Glyoxylate and dicarboxylate metabolism | 1 | 0.458 | 0.000 | Glyceric acid cpd:C00258 |
| Glycerolipid metabolism | 1 | 0.458 | 0.105 | Glyceric acid cpd:C00258 |
| Pyrimidine metabolism | 2 | 0.417 | 0.034 | Uridine cpd:C00299; Deoxyuridine cpd:C00526 |
| Porphyrin and chlorophyll metabolism | 1 | 0.250 | 0.000 | L-Glutamic acid cpd:C00025 |
| Drug metabolism-other enzymes | 1 | 0.235 | 0.056 | 5-Fluorodeoxyuridine cpd:C11736 |
| Amino sugar and nucleotide sugar metabolism | 1 | 0.134 | 0.082 | N-Acetylmannosamine cpd:C00645 |
| Valine, leucine and isoleucine degradation | 1 | 0.126 | 0.012 | 4-Methyl-2-oxopentanoate cpd:C00233 |
| Aminoacyl-tRNA biosynthesis | 2 | 0.119 | 0.000 | L-Threonine cpd:C00188; L-Glutamic acid cpd:C00025 |
| Tryptophan metabolism | 1 | 0.119 | 0.000 | Oxoadipic acid cpd:C00322 |
| Tyrosine metabolism | 1 | 0.088 | 0.071 | 4-Hydroxyphenylpyruvic acid cpd:C01179 |

Hits is the number of significantly differential metabolites in one pathway.

Raw *P* is *P* value calculated from the pathway enrichment analysis.

Impact represents impact value in the pathway topology analysis.
